# Reduced Plasma Orexin-A Concentrations are Associated with Cognitive Deficits in Anorexia Nervosa

**DOI:** 10.1038/s41598-019-44450-6

**Published:** 2019-05-27

**Authors:** Trevor Steward, Gemma Mestre-Bach, Roser Granero, Isabel Sánchez, Nadine Riesco, Cristina Vintró-Alcaraz, Sarah Sauchelli, Susana Jiménez-Murcia, Zaida Agüera, Jose C. Fernández-García, Lourdes Garrido-Sánchez, Francisco J. Tinahones, Felipe F. Casanueva, Rosa M. Baños, Cristina Botella, Ana B. Crujeiras, Rafael de la Torre, Jose M. Fernández-Real, Gema Frühbeck, Francisco J. Ortega, Amaia Rodríguez, José M. Menchón, Fernando Fernández-Aranda

**Affiliations:** 1 Department of Psychiatry, Bellvitge University Hospital-IDIBELL, Barcelona, Spain; 2 Ciber Fisiopatologia Obesidad y Nutrición, Instituto Salud Carlos III (Spain), Madrid, Spain; 3 Department of Clinical Sciences, School of Medicine, University of Barcelona, Barcelona, Spain; 4 Departament de Psicobiologia i Metodologia, Universitat Autònoma de Barcelona, Barcelona, Spain; 5 Unidad de Gestión Clínica de Endocrinología y Nutrición, Instituto de Investigación Biomédica de Málaga (IBIMA), Universidad de Málaga, Hospital Clínico Virgen de la Victoria, Málaga, Spain; 6 Molecular and Celular Endocrinology, Instituto de Investigacion Sanitaria (IDIS), Complejo Hospitalario Universitario de Santiago (CHUS) and Santiago de Compostela University (USC), Santiago de Compostela, Spain; 7 Department of Psychological, Personality, Evaluation and Treatment of the University of Valencia, Valencia, Spain; 8 Integrated Pharmacology and Systems Neurosciences Research Group, Neuroscience Research Program Organization IMIM (Hospital del Mar Medical Research Institute), Barcelona, Spain; 9 Department of Health and Experimental Sciences, Universitat Pompeu Fabra Barcelona, Barcelona, Spain; 10 Department of Diabetes, Endocrinology and Nutrition, Institut d’Investigació, Biomèdica de Girona (IdIBGi), Hospital Dr Josep Trueta, Girona, Spain; 11 Metabolic Research Laboratory, Clínica Universidad de Navarra, University of Navarra-IdiSNA, Pamplona, Spain; 12 CIBER Salud Mental, Instituto Salud Carlos III (Spain), Madrid, Spain

**Keywords:** Psychiatric disorders, Endocrine system and metabolic diseases, Cognitive neuroscience

## Abstract

Orexins/hypocretins are neuropeptides implicated in numerous processes, including food intake and cognition. The role of these peptides in the psychopathology of anorexia nervosa (AN) remains poorly understood. The aim of the current study was to evaluate the associations between plasma orexin-A (OXA) concentrations and neuropsychological functioning in adult women with AN, and a matched control group. Fasting plasma OXA concentrations were taken in 51 females with AN and in 51 matched healthy controls. Set-shifting was assessed using the Wisconsin Card Sorting Test (WCST), whereas decision making was measured using the Iowa Gambling Task (IGT). The AN group exhibited lower plasma OXA levels than the HC group. Lower mean scores were obtained on the IGT in AN patients. WCST perseverative errors were significantly higher in the AN group compared to HC. In both the AN and HC group, OXA levels were negatively correlated with WCST non-perseverative errors. Reduced plasma OXA concentrations were found to be associated with set-shifting impairments in AN. Taking into consideration the function of orexins in promoting arousal and cognitive flexibility, future studies should explore whether orexin partly underpins the cognitive impairments found in AN.

## Introduction

Anorexia nervosa (AN) is a psychiatric disorder characterized by low body weight, severely restricted eating behavior, an extreme pursuit of thinness and an intense fear of weight gain^1^. In addition to the above-mentioned symptoms, patients with AN have repeatedly been found to present altered performance on neuropsychological tasks examining decision-making^2,3^, set-shifting^4^, central coherence^5^, and delay discounting^6,7^. There is also strong evidence to support that alterations in reward processing in AN patients are underpinned by altered reactivity in striatal regions^8^ and to the possibility of hypothalamic inputs being overridden by top-down emotional-cognitive control regions^9^. Additionally, innovative new lines of research suggest that increased activations in fronto-striatal circuits are strongly associated with the maintenance of restrictive eating habits in AN patients^10^. These deficits in executive functioning are thought to contribute to the severity of the disorder and hinder the effectiveness of treatment by encouraging reduced food intake, body image distortions, and rigid thinking styles^11^. Indeed, recent findings indicate that the undernourished state of AN may amplify the tendency to forgo immediate rewards in favor of longer-term goals, thereby perpetuating the symptoms of AN^12^.

Orexin-A (OXA), also known as hypocretin 1, is a neuropeptide synthesized in the hypothalamus that is implicated in the regulation of an array of complex behaviors, including sleep/wakefulness, reward, food intake, and cognition^13–16^. Although OXA neurons were until recently thought to be restricted to the lateral hypothalamus, studies have found that orexinergic fibers are extensively distributed in numerous brain regions, including the posterior hypothalamus and perifornical areas^17^. Orexins are increasingly understood to play a significant role in the pathology of psychiatric disorders^18^, narcolepsy-cataplexy^19^, neurodegenerative diseases, such as Alzheimer’s disease^20^, and in controlling energy homeostasis^21,22^. In the specific case of AN, results thus far have been inconsistent with studies finding plasma OXA levels in patients with AN to be higher^23^, lower^24^, or not significantly different in comparison to healthy controls (HC)^25^. Though few longitudinal studies have carried out in AN patients, it appears that OXA levels decrease following weight restoration^23,24^. However, it must be noted that these studies had relatively small sample sizes and did not follow patients for a long enough time span for full recovery to be reached. OXA levels in AN patients may also potentially be influenced by the diurnal intermittent fasting that is characteristic of the disorder^26^. Such fluctuations in OXA concentrations could have downstream effects on wakefulness^14^ and response to salient cues^27^, thereby influencing executive functioning.

Orexins are believed to be involved in higher cognitive functions due to their role in maintaining the excitability of pyramidal neurons in the prefrontal cortex^18^. One study determined elevated orexins in female rats following repeated exposure to stress to be linked to stress-induced cognitive deficits in the side reversal task of Attentional Set Shifting Test^28^. Likewise, the preclinical literature has consistently suggests that orexins enhance cognitive function, whereas orexin antagonists lead to impairments in different types of cognitive function, such as attention, spatial memory, and social memory^29^. Taking into consideration the aforementioned importance of cognition in treatment response, it would be of great interest to determine the associations between neurocognitive performance and OXA levels in patients with AN. Such findings could shed light on the emerging role of the orexinergic system in the pathophysiology of AN^30^.

## Methods

### Participants and procedure

Participants in this study included 51 women with AN (BMI < 18.5 kg/m^2^) and 51 matched female healthy controls (HC; BMI = 18.5–24.9 kg/m^2^). 65.8% of the sample included in this study was featured in Sauchelli *et al*.^25^. AN participants were consecutively recruited from the Department of Psychiatry at Bellvitge University Hospital (Barcelona, Spain) and were diagnosed by means of structured face-to-face interview, following DSM-5 criteria^31^. All diagnoses were established using an adapted version of the structured clinical interview for DSM-IV (SCID-I)^32^.

Participants were excluded if they were under 18 or over 60 years of age. Males were excluded from the study given the low prevalence of male AN patients in our sample. Several centers belonging to our Spanish Research Network (CIBEROBN) participated in this project. The HC group was a convenience sample (matched for age and education level) recruited via word-of-mouth and advertisements. To be eligible for the HC group, participants could not have a lifetime history of an eating disorder and current obesity.

As described previously^16,25^, clinical and physical assessments were conducted by experienced psychologists, psychiatrists and endocrinologists. Self-report questionnaires were completed as part of the evaluation process at the start of treatment. Patients with AN were voluntarily undergoing day-hospital treatment and all measures were collected upon admission to treatment. All participants completed the same self-reported questionnaires to coincide with the blood sample extraction. Anthropometric information was obtained using a bioimpedance scale.

All participants provided signed informed consent. The University Hospital of Bellvitge Ethics Committee of Clinical Research, the Hospital Universitari de Girona Doctor Josep Trueta Comitè Ètic d’Investigació Clínica, the Parc de Salut Mar Comitè Ètic d’Investigació Clínica, the Hospital Universitario Virgen de la Victoria Subcomisión de Investigación Clínica, Universidad de Navarra Comité de Ética de la Investigación, and the Comissió Deontológica de la Universitat Jaume 1 approved the study, conducted in accordance with the Declaration of Helsinki.

### Measures

#### Orexin-A plasma concentrations

Peripheral blood samples were collected from all participants after an overnight fast. Blood was drawn from an antecubital vein using a 10 mL ethylenediamietraacetic acid (EDTA) containing BD Vacutainer® tube. Samples were centrifuged at 3130 × g for 15 min at 4 degrees Celsius (°C). Plasma and serum were distributed in aliquots and stored at 80 (°C) until analysis. Several plasma bio-chemical variables were measured in duplicate. OXA/hypocretin-1 was measured using a Phoenix Pharmaceuticals Inc. EIA kit (Burlingame, California, USA). Multiple studies have demonstrated that it features a wide detection range, between 0–100 ng/mL, with a linear range of 0.2–2.66 ng/mL, a sensitivity of 0.2 ng/ml, an intra-assay variation <10%, and an inter-assay variation <15%. Our data provide a linear range of 0.30–3.08 ng/mL, a detection limit of 0.16 ng/ml, an intra-assay variation of 9.52% and an inter-assay variation of 13.70%. With respect to its specificity, this kit does not have cross-reactivity with OXA (16–33), orexin B (human), agouti-related protein (83–132)-amide (human), neuropeptide (human, rat), α-MSH (human, rat, mouse) and leptin (human). We did not analyze cross-reactivity with any other molecule.

#### Anthropometric measures

A Tanita BC-420MA was utilized to measure body composition and to calculate BMI. This noninvasive and validated device (Tanita BC-420MA) uses bioelectrical impedance analyses to obtain weight and body composition variables^33^. Height was taken via a stadiometer.

#### The Wisconsin card sorting test (WCST)

The WCST^34^ is a widely used measure to assess an individual’s capacity to plan, control impulsive responses, and be flexible under changing conditions. During this task, participants must match a target card with one of four category cards. After each trial, feedback is given to the participant, indicating whether they have matched the card appropriately. However, the classification rule for matching cards with categories changes during the task and participants must shift and update their previous strategy to perform well on the task. The test ends when the participant has completed six categories or 128 trials. A computerized version of the task was used for this study^35^.

#### The iowa gambling task (IGT)

The IGT^36,37^ is a commonly used measure of decision-making ability. During this task, participants must choose from four decks of cards (A, B, C and D) and each time the participant selects a deck, a specified amount of play money is awarded. The interspersed rewards among theses decks feature probabilistic punishments (monetary losses with varying amounts). Decks A and B are not advantageous as losses are higher than gains; whereas, decks C and D are advantageous since the punishments are smaller. This test is scored by subtracting the number of cards selected from decks A and B from the number of cards selected from decks C and D. Higher scores demonstrate better performance on the task while negative scores are indicative of choosing disadvantageous decks.

#### General psychopathology

The Symptom Checklist-revised (SCL-90-R)^38^ was used to explore general psychopathology. This 90-item, self-report questionnaire is answered on a five-point Likert scale. The items are grouped into nine primary symptom dimensions and it provides a global severity index (GSI) to indicate overall distress. The test has been validated in a Spanish population^39^, with a mean internal consistency of 0.75 (alpha coefficient).

#### Eating disorder symptomatology

The Eating Disorder Inventory-2 (EDI-2)^40^ was used to examine eating disorder severity. This test, consisting of 91 items, each rated on a six-point Likert scale, evaluates eleven cognitive and behavioral characteristics observed in people with eating disorders. A total score is also provided to report overall eating disorder severity. This instrument has been validated in Spanish-speaking populations^41^ with a mean internal consistency of 0.63 (alpha coefficient).

### Statistical analysis

Statistical analysis was carried out with Stata15 for Windows. Comparisons of means between groups (AN and HC) was based on analysis of variance (ANOVA). Associations between neuropsychological variables and orexin levels were estimated with correlation coefficients. In this study, due to the strong association between neuropsychological performance with age and education levels, these two variables were defined as covariates in the statistical analysis. Effect size was measured through Cohen’s-*d* coefficient for mean differences (|*d*| > 0.2 was considered low effect size, |*d*| > 0.5 was considered moderate and |*d*| > 0.8 was considered large)^42^. For the correlation estimates, and due to the strong association between this model and sample size, relevance was based on coefficient measures (|*R*| > 0.10 was considered low effect size, |*r*| > 0.24 was considered moderate and |*r*| > 0.37 was considered large)^43^. Finner’s method controlled for increases in Type-I error due to multiple statistical comparisons (this procedure is included in the Family-wise error rate stepwise procedures and provides a more powerful test than Bonferroni correction)^44^.

## Results

### Sample description and comparison between OXA, body composition and neuropsychological measures

Table 1 contains a description of the study variables, as well as comparisons between the groups. As mentioned above, HC were chosen to match their AN counterparts with respect to age and years of education and no differences were found between groups in these variables (p = 0.666 and p = 1.000, respectively). As expected, patients with AN exhibited significantly higher levels of psychopathology as measured by the SCL-90-R GSI score and higher levels of total eating disorder severity on the EDI-2.

**Table 1:** Sample description and comparison between groups in orexin levels, body composition and neuropsychological performance.

	Anorexia (*n* = *51)*		Control (*n* = *51)*			
	*Mean*	*SD*	*Mean*	*SD*	*p*	*|d|*
Age (years)	27.43	8.65	26.75	7.32	0.666	0.09
EDI-2 total score	78.78	43.72	22.59	16.91	**0**.001*	**1**.**70**^**†**^
SCL-90-R GSI	1.48	0.74	0.56	0.39	**0**.001*	**1**.**56**^**†**^
Plasma orexin-A levels (pg/ml)	2.43	0.82	2.99	0.67	**0**.001*	**0**.**75**^**†**^
BMI (kg/m2)	17.11	1.27	21.37	1.81	**0**.001*	**2**.**73**^**†**^
Body fat mass (%)	6.37	3.11	15.32	4.42	**0**.001*	**2**.**34**^**†**^
^1^IGT Block 1 (raw score)	−1.95	4.20	−2.44	7.48	0.714	0.08
^1^IGT Block 2 (raw score)	−0.77	4.41	5.69	8.35	**0**.001*	**0**.**97**^**†**^
^1^IGT Block 3 (raw score)	−0.20	5.36	7.01	8.48	**0**.001*	**1**.**02**^**†**^
^1^IGT Block 5 (raw score)	0.42	6.69	9.30	9.28	**0**.001*	**1**.**10**^**†**^
^1^IGT Block 6 (raw score)	0.79	9.26	6.80	10.61	**0**.007*	**0**.**60**^**†**^
^1^IGT Total (raw score)	−1.71	21.06	26.36	27.82	**0**.001*	**1**.**14**^**†**^
^1^WCST Trials (raw score)	92.51	18.05	86.96	16.32	0.182	0.32
^1^WCST Correct (raw score)	65.67	12.39	66.98	6.92	0.547	0.13
^1^WCST Errors (raw score)	26.91	17.19	19.99	17.52	0.152	0.40
^1^WCST perseverative errors (raw score)	13.57	9.87	9.53	5.22	**0**.045*	**0**.**51**^**†**^
^1^WCST non-perseverative errors (raw score)	13.33	16.99	10.46	11.41	0.350	0.20
^1^WCST conceptual (raw score)	57.90	18.90	61.76	11.33	0.250	0.25

*Note*. ^1^Results adjusted for age and education level. SD: standard deviation.

*Bold: significance (0.05 level). ^†^Effect size in the moderate (|*d*| > 0.50) to high range (|*d*| > 0.80).

*p*-value includes Finner’s correction for multiple statistical comparisons.

EDI-2: The Eating Disorder Inventory-2; SCL-90-R: The Symptom Checklist-revised; BMI: body mass index; IGT: Iowa Gambling Task; WCST: Wisconsin Card Sorting Test.

Table 1 also contains the results of the ANOVA, adjusted for age and education level, comparing the mean scores between the groups. The AN group exhibited lower BMI and body fat mass % than the HC group. Lower means were obtained on all the IGT measures (except for block 1) in AN patients. WCST perseverative errors were significantly higher in the AN group compared to HC.

### Correlation analysis

**Figure 1:**
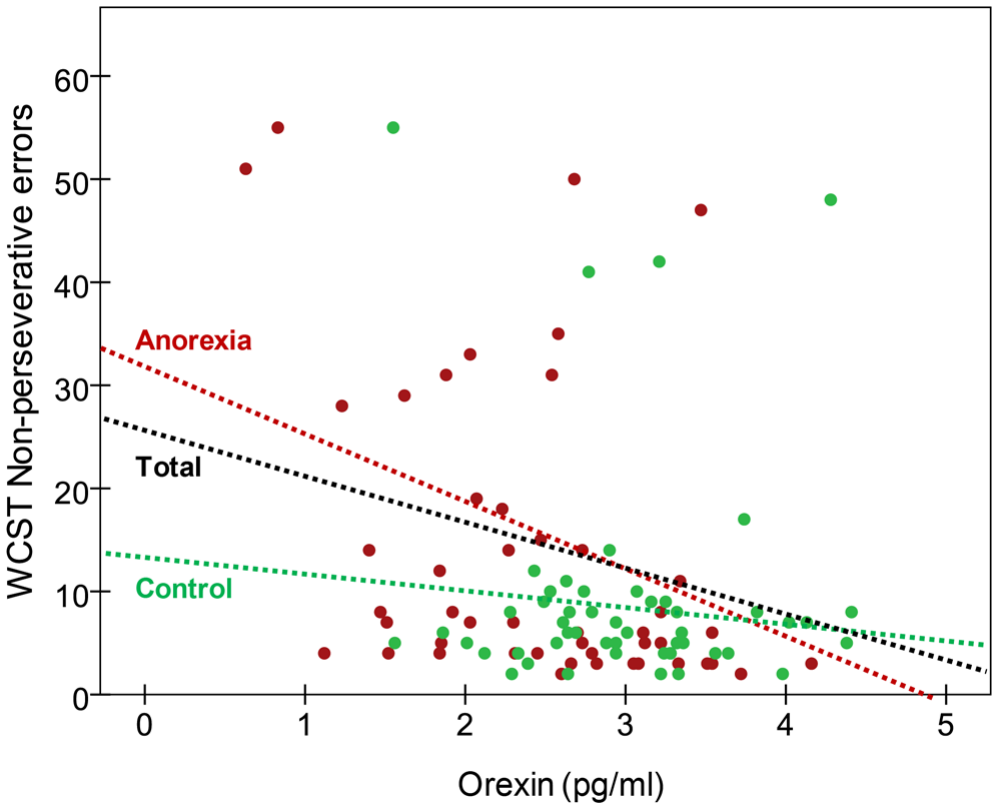
Scatterplot for orexin-A plasma levels (pg/ml) with WCST non-perseverative errors.

Table 2 contains the partial correlation matrix (coefficients adjusted for age, education and BMI) measuring the association between the neuropsychological measures and plasma OXA levels. Taking into account previous studies indicating an association between clinical state and neuropsychological performance^2^, we chose to run our correlational analyses including BMI as a covariate. Negligible differences in the results were found when BMI was not included as a covariate. In the AN group, OXA levels were negatively correlated with IGT block 1 performance, WCST number of trials, WCST errors and WCST non-perseverative errors. In the HC group, OXA levels were positively correlated with IGT block 4 scores and negatively correlated with WCST non-perseverative errors. Figure 1 presents a scatterplot of OXA levels and WCST non-perseverative errors in both groups and the whole sample. Regarding the potential associations between the measures of ED severity (EDI-2 total raw scores) and psychopathology (SCL-90R GSI) with OXA levels, the bottom of the Table 2 contains these partial correlations, whose effect sizes were in the low/null range.

**Table 2:** Associations between neuropsychological measures with orexin levels (pg/ml): partial correlations (adjusted for age, education level and BMI).

	Anorexia *(n* = *51)*	Control *(n* = *51)*
IGT Block 1 (raw score)	**−0**.**331**^**†**^	−0.097
IGT Block 2 (raw score)	0.089	0.142
IGT Block 3 (raw score)	0.104	0.205
IGT Block 4 (raw score)	0.080	**0**.**241**^**†**^
IGT Block 5 (raw score)	0.136	0.175
IGT Total (raw score)	0.060	0.226
WCST Trials	**−0**.**300**^**†**^	−0.206
WCST Correct	−0.041	0.088
WCST Errors	**−0**.**241**^**†**^	−0.227
WCST perseverative errors	0.014	−0.172
WCST Non-perseverative errors	**−0**.**371**^**†**^	**−0**.**246**^**†**^
WCST Conceptual	0.126	0.200
EDI-2 total score	0.068	0.021
SCL-90-R GSI	0.078	−0.027

*Note*. ^†^Effect size in the moderate (|*r*| > 0.24) to high range (|*r*| > 0.37).

IGT: Iowa Gambling Task; WCST: Wisconsin Card Sorting Test.

## Discussion

In this study, we examined the association between OXA levels and cognitive impairments in patients with AN. Our findings indicate that OXA levels in AN patients were significantly lower than in HCs, though, it should be noted that diverging results in other AN samples have been found^23^. These discrepancies could stem from the numerous differences in the characteristics of the patients featured in the study at hand and other studies. For example, our study included patients with both AN restrictive subtype and binge-eating/purging subtype. One study utilizing an animal model of binge eating found that orexin antagonists reduced binge eating episodes preceded by stress and dietary restriction, suggesting that orexin could play distinctive role in AN patients that binge eat^45^. Other potential confounding factors include the effects of intermediate fasting^26^, sleep patterns^46^, and pharmacological treatment^47^. Recent research has also illustrated how insufficient up-regulation of orexin (Hcrt) genes during activity-based anorexia contributes to greater suppression of food intake and greater body weight loss in prenatal stress (PNS) exposed rats. This suggests that OXA may be useful for the discovery of biomarkers of weight loss vulnerability in order to gain a better understanding of neural circuits implicated in AN pathology^30,48^. Still, longitudinal studies are needed to determined whether such alterations in OXA are present before the onset of AN and after long-term recovery.

We also identified a significant correlation between non-perseverative errors on the WCST and orexin levels in both the AN and HC group. Non-perseverative errors are indicative of an inability to discern patterns and detect favorable choice. Our finding dovetails with the results of a recent study demonstrating that intra-basal forebrain infused OXA facilitated both the acquisition and reversal portions of an olfactory discrimination task^49^. Similarly, increased levels of OXA have been linked to fewer perseverative errors and reduced negative symptoms in patients with schizophrenia^50^ compared to patients with lower orexin levels. Taking into consideration the function of orexins in promoting arousal and responding to stress, it could be posited that reduced OXA activity could partly underpin the cognitive impairments found in AN^51,52^.

In line with past research, we also identified decision-making deficits in patients with AN using the IGT^2,3^. Poor decision-making is known to be more pronounced during the acute phase than in the recovered state of AN^2^ and it would be of interest to explore whether such improvements coincide with changes in OXA levels. In the study at hand, OXA levels only negatively correlated with scores from the first block of the task in AN patients, but not with overall performance. Other studies have attempted to use intranasal administration of orexin peptides for age-related cognitive dysfunction^53^ and it may be of clinical interest to explore the possibility of using OXA to potentially offset the cognitive deficits found in certain psychiatric disorders^54^ or accompanying aging^55^. However, more basic research would be needed before such an endeavor could be undertaken.

This study must be interpreted in light of its limitations. For instance, diverging reports of increased or decreased levels of OXA in AN may result from sample heterogeneity (i.e. AN subtype, age and weight status). Another potential limitation of our study is the use of measured plasma OXA levels as a proxy for central orexin system activity. There is evidence in rodent studies to infer that OXA crosses the blood brain barrier^56,57^ and that the OXA plasma levels in humans are indicative of peptide release from both the brain and the gut^58^. However, no direct measure of CNS orexin levels were taken due to the high levels of invasiveness this procedure would entail. The kit used in this study could have other cross-reactivity than those mentioned by manufacturer. In this sense, we have not analyzed cross-reactivity with other molecules. However, our study does not intend to analyze this factor nor be a validation study of a kit that is already on the market and that has been widely used in other studies. Furthermore, no conclusions regarding causality can be made from our findings given the cross-sectional nature of our study. There is previous research to support that orexin levels could may in fact further decrease with treatment^23,24^, and it would be useful to examine orexin levels in individuals who have obtained full recovery from AN. Lastly, recent work has identified that orexins are regulators of sex differences in the behavioral adaptations to- and consequences of repeated stress^28^. Therefore, it would be beneficial for future studies to assess sex-related differences in orexin system functioning in patients with AN.
